# Topology Optimization of High‐Performance Optomechanical Resonator

**DOI:** 10.1002/advs.202512534

**Published:** 2025-11-26

**Authors:** Yincheng Shi, Fengwen Wang, Dennis Høj, Ole Sigmund, Ulrik Lund Andersen

**Affiliations:** ^1^ Center for Macroscopic Quantum States (bigQ) Department of Physics Technical University of Denmark Fysikvej Kgs. Lyngby 2800 Denmark; ^2^ Department of Civil and Mechanical Engineering Technical University of Denmark Koppel's Allé 404 Kgs. Lyngby 2800 Denmark

**Keywords:** finite element analysis, nanofabrication techniques, opto‐mechanical resonator, topology optimization

## Abstract

High quality mechanical resonators are critical for driving advances in quantum information technologies, precision sensing, and optomechanics. However, achieving compact resonator designs that maintain high performance is a key challenge. In this study, a new class of compact resonators optimized to operate at higher‐order eigenmodes is presented, achieving both high frequencies and enhanced quality factor‐frequency (*Qf*) products. By employing topology optimization to maximize the damping dilution factor, these resonators achieve minimized edge bending losses and enhanced intrinsic damping. Their high‐(*Qf*) performance and compact form factor position these resonators as promising candidates for applications in quantum information transduction, advanced optomechanical systems, and next‐generation sensing technologies.

## Graphical abstract

Compact mechanical resonators designed to operate at higher‐order modes are presented, achieving high frequencies and enhanced quality factor‐frequency (*Qf*) products. Using topology optimization, edge losses are reduced and damping is improved. These resonators offer strong performance in a small footprint, making them ideal for quantum transduction, optomechanics, and advanced sensing applications.

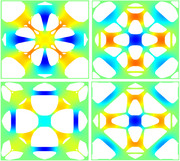

## 1 Introduction

High‐Q mechanical resonators are crucial for precision sensing, spin detection, and emerging quantum technologies, including transducers, quantum memory, and phononic computing.^[^ ^1^, ^2^, ^3^, ^4^ ^]^

Ultra‐high quality factors—exceeding 100 000 times the intrinsic material limits of the mechanics^[^ ^5^, ^6^ ^]^—can be achieved through dissipation dilution, first observed in gravitational‐wave detectors^[^ ^7^ ^]^ and later in Si_3_N_4_ nano‐strings.^[^ ^8^ ^]^ It arises from tensile strain and geometric optimization, allowing elastic energy to be stored in tension rather than bending, reducing losses and enhancing *Q*. The dilution factor is *D*_*q*_ = *Q*/*Q*_0_, with *Q*_0_ the intrinsic quality factor.^[^ ^5^, ^9^ ^]^

A basic understanding of damping dilution can be gained through models of pre‐stressed strings and membranes.^[^ ^5^ ^]^ These models show that the dilution factor *D*_*q*_ is positively correlated with the aspect ratio (length‐to‐thickness) and strain, achievable through nanofabrication techniques like LPCVD deposition of pre‐stressed Si_3_N_4_ thin films. Additionally, *D*_*q*_ is inversely proportional to edge bending terms and inversely quadratic with respect to anti‐nodal bending terms. By minimizing edge bending, or “soft clamping,” *D*_*q*_ can be significantly enhanced. This technique, initially demonstrated with phononic crystals (PnCs) featuring central defects,^[^ ^10^ ^]^ has been refined with strain engineering^[^ ^11^, ^12^ ^]^ and mass engineering.^[^ ^13^ ^]^ Other methods, including fractal‐like^[^ ^14^ ^]^ and hierarchical^[^ ^15^ ^]^ structures, as well as perimeter mode designs,^[^ ^16^ ^]^ have led to advanced resonator configurations that effectively minimize edge bending.

While analytical models provide insight into simple geometries, they often fall short in accounting for the complex geometry of advanced resonator designs, particularly those using phononic crystals. For such structures, numerical simulations are essential. Various optimization strategies have been employed to enhance resonator performance, including tuning the sizes of PnC cells and defects,^[^ ^11^ ^]^ experimenting with different PnC configurations,^[^ ^10^ ^]^ refining clamping geometries,^[^ ^17^ ^]^ and systematically adjusting parameters across the resonator.^[^ ^18^ ^]^ These approaches, often starting from heuristic design estimates and sometimes enhanced with machine learning,^[^ ^19^ ^]^ have yielded resonators with exceptional *Q* and *Qf* products.

Despite these achievements, unexplored designs with potentially superior performance may remain undiscovered. This limitation arises from two main issues. First, the initial predefined geometries typically constrain the optimization to a few variables, such as width, length or radius of curvature, restricting the scope of potential design space. Second, the gradients of *Q* or *Qf* with respect to these variables are often difficult to calculate, making many well‐established gradient‐based optimization algorithms unsuitable for this problem.

These challenges can be tackled using methods like topology optimization,^[^ ^20^ ^]^ a numerical approach that seeks configurations with optimized performances without requiring a predefined geometry. It allows the topology of the structure to evolve throughout the optimization process, accommodating significant design changes. Moreover, topology optimization can be solved with various gradient‐based algorithms. Recent studies^[^ ^21^, ^22^ ^]^ have applied this method, using finite element analysis (FEA)^[^ ^23^, ^24^ ^]^ to evaluate *Q* at the fundamental eigenmode as the optimization objective. The resulting optimized designs exhibit features similar to trampoline resonators^[^ ^18^ ^]^ near the central pad, validating the design approach, while also introducing novel structural details near the outer boundary. Compared to similar dimensioned trampolines^[^ ^18^ ^]^ these optimized structures achieve significantly improved *Qf* products, enabling the resonators to meet the demanding requirement of *Qf* > 6 × 10^12^Hz at room temperature.^[^ ^21^, ^22^ ^]^

Most studies focus on the fundamental mode,^[^ ^18^, ^21^, ^22^ ^]^ sometimes within a PnC band gap.^[^ ^6^, ^11^, ^13^ ^]^ However, exploring higher‐order modes can be valuable. One reason is that achieving the required *Qf* threshold to enter the quantum regime at room temperature necessitates balancing *Q* and *f*, a trade‐off that topology optimization can address. Additionally, higher‐order modes exhibit more intricate mode profiles than the fundamental mode, potentially reducing edge bending near clamping boundary without needing a phononic crystal structure. This could lead to more compact resonator designs.

In this study, the resonator is designed for application in optical cavities, potentially using a membrane‐in‐the‐middle configuration. Therefore, the resonator is two‐dimensional (2D) with a central pad, enabling coupling with a laser through radiation pressure forces.^[^ ^25^ ^]^ Only intrinsic damping, primarily due to surface losses,^[^ ^26^ ^]^ is considered and is quantified by the dilution factor *D*_*q*_. External losses, such as gas damping, are mitigated by placing the resonator in a high‐quality vacuum environment. In the finite element analysis (FEA), edge bending caused by high pre‐stress^[^ ^5^ ^]^ is taken into account, and a locally refined mesh is used to capture this effect accurately. Additionally, we explore the topology optimization of a resonator targeting higher‐order eigenmodes, with *D*_*q*_ as the optimization objective.

## 2 Results

### 2.1 Numerical Simulation and Optimization

**Figure 1:**
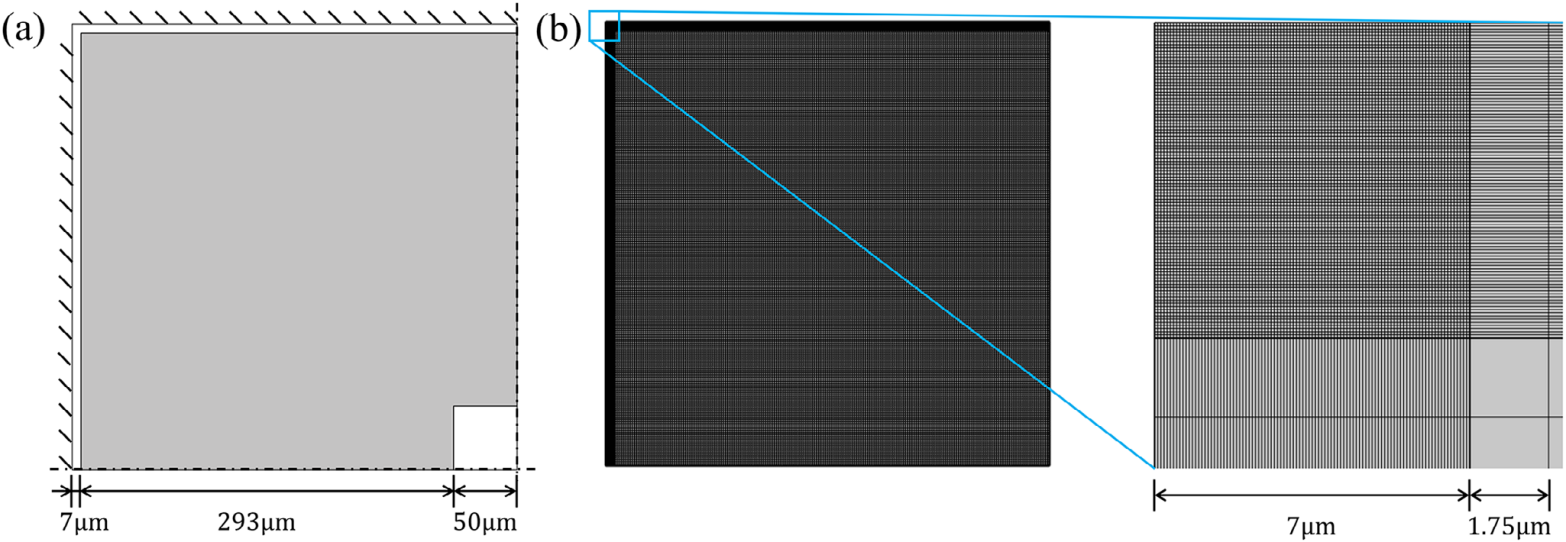
Specifications of numerical model. a) Dimensions and boundary conditions: The left and upper boundaries are clamped while the other two are set as symmetric conditions. The gray part represents the design domain, while the white regions indicate the non‐design domain. b) Mesh details: The complete mesh (left) and the locally refined mesh near the corner point (right). The locally defined region spans 7 μm with 100 elements, each with a resolution of 70nm. The coarser mesh has a resolution of 1.75 μm, resulting in a total mesh size of 296×296 quadrilateral elements.

The numerical model used for finite element analysis and topology optimization is detailed in **Figure** **1**. The resonator is composed of a pre‐stressed Si_3_N_4_ layer with a nominal thickness of 50nm, a Young's modulus of 250GPa, a material density of 3100 kg m^−3^, and a Poisson's ratio of 0.23. The intrinsic quality factor *Q*_0_ is set to 4000 based on reported results.^[^ ^21^ ^]^ To obtain compact device design, the resonator is embedded within a square window of 700 $\times 700\mu m^{2}$. A 7μm rim is included to accommodate variations in window size due to changes in silicon wafer thickness, and a central pad of 100 $\times 100\mu m^{2}$ facilitates coupling between light and mechanical resonator via radiation pressure force.^[^ ^25^ ^]^ The rim and central pad remain fixed during topology optimization while the remaining area serves as the design domain where the design variables are iteratively updated. To optimize computational efficiency, only a quarter of the domain is analyzed, applying the boundary conditions shown in Figure 1a. Due to the high pre‐stress from Si_3_N_4_ deposition, the curvature of mode shape changes abruptly near the fixed boundary^[^ ^5^ ^]^ and thus a locally refined mesh is applied on the rim while a coarser mesh is used for the rest of the domain, as shown in Figure 1(b).

**Figure 2:**
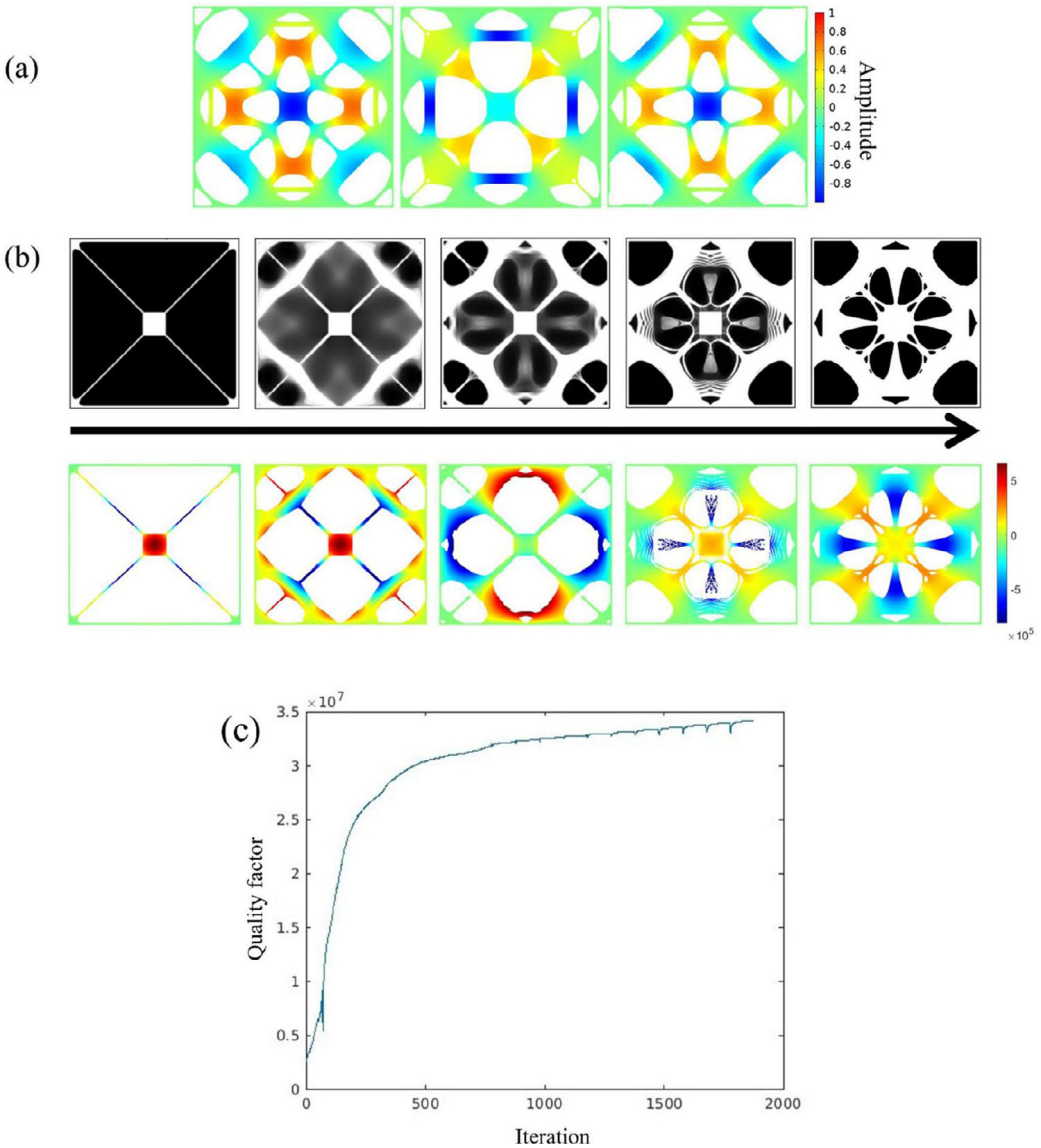
Configurations of optimized resonators. a) Design 2, 3, and 4, showing the normalized targeted eigenmode (4th, 5th, and 4th, respectively) profiles of out‐of‐plane displacement plotted on the smoothed optimized designs. b) Upper: evolution of design 1 from the initial guess to the final optimized design. Black indicates voids, while white represents the Si_3_N_4_ layer. Geometric constraint is applied on the 4th design. In the final optimized structure, small holes were manually filled to facilitate fabrication. Lower: corresponding targeted eigenmodes (4th) plotted on elements with physical design variable ${\bar{\rho }}_{e}>0.65$. c) History of optimization of quality factor. There are spikes during optimization as the projection parameter from the three‐field method is updated every 100 steps.

The intrinsic loss is the only loss mechanism considered in this study, modeled as being proportional to linear strain energy at the targeted eigenmode. To enable effective opto‐mechanical coupling through radiation pressure forces, the central pad must maintain a nonzero out‐of‐plane displacement, as shown in **Figure** **2**a. In some cases, such as in designs 1 and 3, the out‐of‐plane motion of the central pad does not reach the maximum magnitude, but this does not pose a problem for ringdown measurements and subsequent applications. The overall quality factor is estimated as *Q* = *Q*_0_ × *D*_*q*_ where *Q*_0_ is the intrinsic quality factor and *D*_*q*_ is the damping dilution factor. The material distribution is optimized by maximizing *D*_*q*_ via density‐based topology optimization, with a maximum material occupation ratio set to 50%. The frequency of the fundamental mode is constrained to ensure structural connectivity. Details of topology optimization are provided in the Methods section. Different designs are achieved using various initial guesses: Design 1 starts from the nominal design reported by Norte et al.^[^ ^18^ ^]^; Design 2 begins with a homogeneous square membrane. Design 3 uses the same initial guess as Design 1 but is optimized for a higher targeted eigenmode, while Design 4 starts with the same initial guess as Design 2 but with a modified feature selection methods that removes small tethers at the corners and near the central pad. The optimization evolution of Design 1 is shown as an example in Figure 2b, illustrating the progression from the initial guess to the final optimized configuration.

### 2.2 Fabrication and Characterization

**Figure 3:**
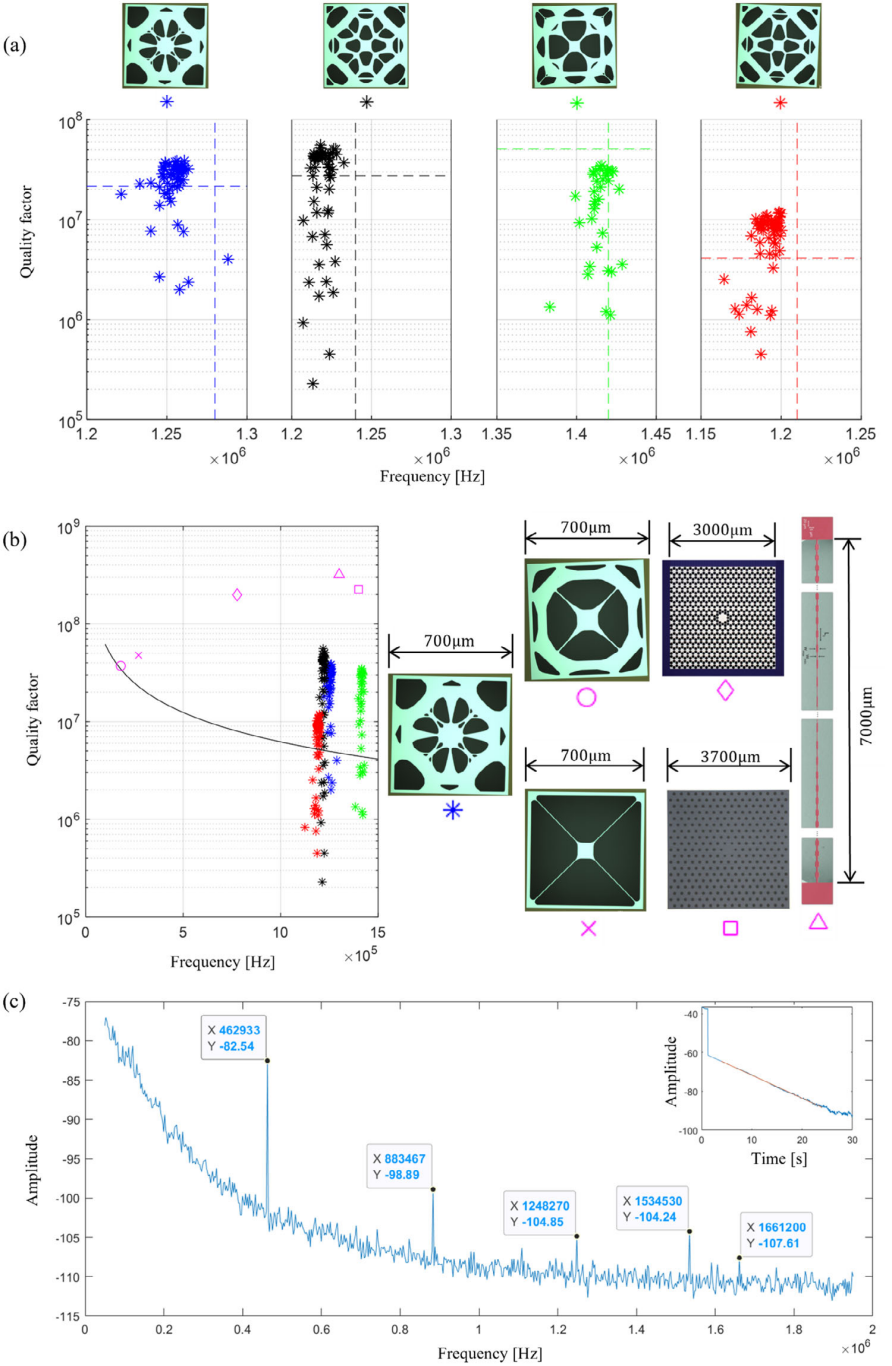
a) Ringdown measured *Q* and *f* values. Optical microscope images for designs 1–4 are shown on top, where the white regions represent the Si_3_N_4_ layer, and black regions indicate voids. Data points correspond to measured values, while the dashed lines represent the numerically calculated *Q* (horizontal) and *f* (vertical) using a body‐fitted mesh in COMSOL Multiphysics, as listed in **Table** **1**. b) Comparison with reported results: *Qf* (left) and size (right). The black solid lines indicates the contour where *Qf*=6.2 × 10^12^. The yield represents the number of samples with *Qf* exceeding this limit out of the total measured samples for each design: 54/58 for Design 1, 48/57 for Design 2, 27/35 for Design 3, and 46/62 for Design 4. The highest measured *Qf* values of the designs are highlighted with magenta circle^[^ ^22^ ^]^ and crosses.^[^ ^18^ ^]^ *Q* values of other designs, indicated by magenta diamond,^[^ ^10^ ^]^ squares^[^ ^13^ ^]^ and triangles^[^ ^11^ ^]^ are scaled to thickness of 50nm for consistency with design 1‐4. c) A representative power spectrum of Design 1. The inset shows the corresponding ringdown curve over 30s at the targeted mode, with *f*: 1.248MHz and *Q*: 29.13× 10^6^.

The optimized structures were patterned on a 50 nm Si_3_N_4_ layer deposited by low pressure chemical vapor deposition (LPCVD) with a pre‐stress of ≈1.2GPa. Both the pre‐stress and thickness exhibited minimal variation due to fluctuations in the deposition process. For comparison, two additional resonator designs from previous studies^[^ ^18^, ^22^ ^]^ were fabricated alongside the optimized structures, as shown in **Figure** **3**b.

Ringdown measurements were conducted in a vacuum system with a pressure below 10^−6^ mbar at room temperature. The measured *Q* and *f* values are presented in Figure 3a. For designs 1–4, more than 75% of the measured samples met the minimum requirement for entering the quantum regime at room temperature, defined by *Qf*>6.2 × 10^12^.^[^ ^25^ ^]^ A power spectrum and the corresponding ringdown curve for Design 1 are shown in Figure 3c. The measured *Q* values display some variation, likely due to contamination by microscopic particles, which can result from cross‐contamination during wet‐chemical processing or exposure during storage. Given that the quality factor due to gas damping is proportional to the operating frequency of the resonator, higher pressure conditions can be tolerated compared to those reported in previous studies.^[^ ^22^ ^]^ With a pressure below 10^−6^ mbar, gas damping is effectively mitigated and, therefore, not included in the numerical model. Another potential loss mechanism is phonon tunneling loss (PTL),^[^ ^5^ ^]^ which arises from energy radiation from the resonator to the substrate. However, this effect was also excluded from the numerical model. The close agreement between the *Q* values measured through ringdown and those predicted by the numerical model indicates that PTL is not a dominant source of loss and can be safely neglected. As shown in Figure 3a, designs 1, 2, and 4 exhibit a slight discrepancy between the numerically estimated *Q* values and those measured through ringdown, where the former should represent an upper bound due to the exclusion of PTL. The numerical estimates assume *Q* = *Q*_0_ × *D*_*q*_, with *Q*_0_ = 4000 based on previously reported values.^[^ ^18^, ^21^, ^22^ ^]^ In reality, *Q*_0_ can vary between 3000 to 7000 across different devices. When the upper bound of *Q*_0_ is considered, the discrepancy between the numerical and experimental results is resolved.

Comparisons of *Qf* product and size between the proposed designs and previously reported results are shown in Figure 3b. The proposed designs demonstrate an increased *Qf* product compared to resonators with similar dimensions. This improvement is primarily due to an increase in the eigenfrequency, while the *Q* factor is either optimized (design 1, 2 and 4) or maintained at a similar level (design 3). In contrast, designs from [10, 11, 13] exhibit higher *Q* and *Qf* values than those of the proposed designs. This advantage stems from the use of phononic crystals, which effectively eliminate edge‐bending losses near the clamping boundaries, a limitation that still affects the proposed designs. Additionally, a larger aspect ratio^[^ ^11^ ^]^ contributes to a higher *Q* factor. Despite these differences, the proposed designs may still be preferred in some scenarios where compact devices are required. Furthermore, in nano‐fabrication, a large aspect ratio significantly increases the complexity and reduces the yield, which could be a critical drawback for large‐scale production or practical applications. Thus, the proposed designs offer a balance between performance and manufacturability, making them advantageous in specific use cases.

## 3 Discussions

Several parameters and figures of merit are numerically evaluated in Table 1 using COMSOL Multiphysics, with the simulation geometry precisely matching that used in fabrication. The proposed resonators, characterized by high *Q* and *f*, offer significant advantages for applications in quantum opto‐mechanics and sensing. Compared to Trampoline 2,^[^ ^18^ ^]^ which has a similar total material occupation but operates at its fundamental mode, the effective mass^[^ ^27^ ^]^ of resonators working at higher order modes is generally larger. Even for the most optimal design among the proposed resonators (Design 2), this increased mass results in less stringent requirements for ground‐state cooling ($\sqrt{S_{xx}^{imp,gs}}=\sqrt{2\hbar^{2}Q_{0}/\left(k_{B}T_{0}\omega m_{eff}\right)}$ and worse force sensitivity ($\sqrt{S_{FF}^{th}}=\sqrt{4k_{B}T_{0}m_{eff}\omega /Q}$) (where ω = 2π*f*, *T*_0_ = 300K, *k*_*B*_ is Boltzmann's constant, and ℏ is the reduced Planck constant). However, our resonators operate at significantly higher eigenfrequencies than both Trampoline 1 and 2. To directly compare performance at similar frequencies, we also evaluated Trampoline 1 and 2 at higher‐order (HO) eigenmodes exhibiting out‐of‐plane motion on the central pad. These modes have eigenfrequencies of 1.15 and 1.27 MHz, respectively, comparable to those of Designs 1–4. In this frequency range, our optimized resonators achieve substantially higher quality factors, confirming that the proposed topology optimization method effectively enhances *Q* at targeted higher‐order modes. Moreover, while the effective mass scales with the total material occupation,^[^ ^27^ ^]^ our topology‐optimized resonators at higher‐order modes achieve lower effective mass than Trampoline 2 despite their much smaller material occupation, highlighting the potential of topology optimization for reducing the effective mass while maintaining high *Q*. In contrast, PnC‐membrane, designed with phononic crystal (PnC) structures, relies on periodic unit cells to engineer band gaps. While this design achieves high quality factors by suppressing boundary losses, it results in a much larger device footprint (≈3000 µm) compared to the compact size of our designs (700 µm). This scale difference leads to an effective mass that is roughly six orders of magnitude higher, which offsets the benefits of a high *Q* and results in inferior ground‐state cooling performance and force sensitivity compared to Design 2. These results highlight that when multiple figures of merit are considered—not just *Q* but also device compactness and effective mass—topology optimization offers significant advantages over conventional PnC‐based approaches.

**Table 1:** Parameters and figures of merit.

	*f* [MHz]	*Q* (× 10^6^)	*m*_*eff*_ [kg]	$\sqrt{S_{xx}^{imp,gs}}$ [$m{\sqrt{\mathrm{Hz}}}^{-1}$]	$\sqrt{S_{FF}^{th}}$ [$N{\sqrt{\mathrm{Hz}}}^{-1}$]
Design 1	1.28	21.55	1.59 × 10^−12^	1.51 × 10^−18^	1.98 × 10^−16^
Design 2	1.24	27.53	1.25 × 10^−12^	1.95 × 10^−18^	1.53 × 10^−16^
Design 3	1.42	51.02	9.74 × 10^−13^	2.81 × 10^−18^	1.06 × 10^−16^
Design 4	1.21	4.12	1.41 × 10^−12^	7.19 × 10^−19^	4.15 × 10^−16^
Trampoline 1^[^ ^22^ ^]^	0.28	29.22	8.20 × 10^−13^	5.22 × 10^−18^	5.72 × 10^−17^
Trampoline 1 ‐ HO	1.15	4.92	5.81 × 10^−13^	3.30 × 10^−19^	9.04 × 10^−16^
Trampoline 2^[^ ^18^ ^]^	0.19	19.77	4.81 × 10^−13^	6.80 × 10^−18^	4.39 × 10^−17^
Trampoline 2 ‐ HO	1.27	0.34	7.90 × 10^−14^	8.51 × 10^−19^	3.50 × 10^−16^
PnC‐membrane^[^ ^10^ ^]^	0.77	214	1.60 × 10^−8^	1.21 × 10^−19^	2.46 × 10^−15^

Previously, high *Qf* resonators^[^ ^21^, ^22^ ^]^ were obtained via topology optimization at fundamental frequencies below 500 kHz. Higher‐frequency resonators were typically realized using phononic crystal methods combined with heuristic optimizations,^[^ ^10^, ^13^ ^]^ which resulted in larger dimensions (∼mm) compared to those obtained through topology optimization (700μm). The approach proposed in this article combines the strengths of both methods, allowing for the specification of any eigenmode, provided that its mode profile supports effective opto‐mechanical coupling, while simultaneously optimizing *Q* at the targeted mode. The resulting resonators exhibit high eigenfrequencies, compact dimensions, and high *Q* factors, along with favorable bending energy distributions, as summarized in **Table** **2**. Notably, Design 3 shows a significantly reduced proportion of edge bending loss—less than 20% of the total bending energy—compared to the other designs, which exhibit ratios similar to Trampoline 1 and 2. This lower edge bending ratio correlates with the highest measured quality factor among the designs (Table 1), consistent with the dominant role of boundary loss in determining *Q*. It should be emphasized that the absolute energy values in Table 2 are dependent on the scaling used in the pre‐stressed eigenfrequency simulations, and therefore not directly comparable across systems; however, the relative energy distribution (i.e., the ratio of edge to total bending energy) provides meaningful insight into the dissipation characteristics. The power spectrum shown in Figure 3c demonstrates that the targeted mode is well isolated from neighbour modes, effectively replicating the band gap behavior achieved with phononic crystal designs. This method also has the potential to extend the targeted mode to even higher frequencies, such as 10MHz, by carefully selecting an appropriate eigenmode profile.

**Table 2:** Bending energy distribution.

	*f* [MHz]	*W*_*b*, *tot*_[*J*]	*W*_*b*, *clamp*_[*J*]^[^ ^28^ ^]^	*W*_*b*, *clamp*_/*W*_*b*, *tot*_
Design 1	1.28	3.00×10^9^	2.21×10^9^	73.67%
Design 2	1.24	2.20×10^9^	1.30×10^9^	59.10%
Design 3	1.42	2.56×10^8^	1.57×10^9^	16.31%
Design 4	1.21	1.41×10^10^	1.17×10^10^	82.98%
Trampoline 1^[^ ^22^ ^]^	0.28	1.08×10^8^	7.50×10^7^	69.44%
Trampoline 2^[^ ^18^ ^]^	0.19	7.00×10^8^	5.06×10^8^	72.29%

The proposed FEA and topology optimization approach is highly adaptable to various extensions. Due to the nature of the opto‐mechanical coupling, the targeted mode always exhibits out‐of‐plane displacement on the central pad. In other applications, such as ultra‐fast force microscopy^[^ ^29^ ^]^ or attonewton‐scale force sensitivity measurements,^[^ ^30^ ^]^ a torsional rotation may be preferred.^[^ ^31^ ^]^ This can be achieved by defining a torsional mode as the targeted in the optimization process. Additionally, other parameters, such as $\sqrt{S_{xx}^{imp,gs}}$ and $\sqrt{S_{FF}^{th}}$ can be incorporated as constraints in the optimization model to tailor performance further.

Currently, the optimization focuses solely on the geometry of the resonator. However, other factors, such as variations in material properties or crystal orientations during fabrication, could be included by introducing additional sets of design variables. Furthermore, this model could be integrated with phononic crystal methods, for example, by optimizing the geometry or distribution of phononic crystal cells operating at different frequencies, or by refining the geometry of defects within the crystal structure. This hybrid approach could further enhance the performance and versatility of the resonator designs.

## 4 Experimental Section

### Finite Element Analysis

Finite element analysis (FEA) was used to solve the pre‐stressed resonator model. To prevent shear‐locking in the extremely thin membrane, quadrilateral MITC4 (Mixed Interpolation of Tensorial Components) cell elements were employed.^[^ ^32^ ^]^ Moreover, to suppress instabilities in the eigenfrequency analysis, the translational displacements were interpolated using quadratic shape functions, while the rotational displacements were interpolated using linear shape functions. The high pre‐stress resulting from the Si_3_N_4_ layer deposition caused abrupt changes in the curvature of the eigenmode profile near the fixed boundaries.^[^ ^5^ ^]^ To accurately capture these localized features, a locally refined mesh was employed in the regions near the fixed boundaries.

The FEA process was composed of two steps. The first step was a linear static analysis to determine the distribution of pre‐stress once the resonator is released. The second step was an eigenvalue analysis that incorporated the pre‐stress,[^[^ ^21^, ^33^ ^]^ solving for the targeted eigenfrequency and the corresponding eigenmode profile:

(1) $$K_{0}U_{0}=F_{0}\left(\sigma_{0}\right)$$

(2) $$\left(K_{0}+K_{\sigma }\left(U_{0}\right)-\omega_{j}^{2}M\right)\phi_{j}=0$$

Here, $K_{0}$ is the linear system stiffness matrix, $F_{0}$ is the load vector derived from the initial stress, $K_{\sigma }$ is the stress stiffness matrix dependent on the initial stress $\sigma_{0}$ and the displacement vector $U_{0}$, and M represents the consistent mass matrix of the system. The terms ω_*j*_ and $\phi_{j}$ correspond to the eigenfrequency and eigenmode profile of the *j*th mode, which is the targeted mode. The quality factor, *Q*, is defined as *Q* = *Q*_0_*D_*q*_, where *Q*_0_ = 4000 is the intrinsic quality factor and *D*_*q*_ is the damping dilution factor,^[^ ^5^ ^]^ calculated at the *j*th eigenmode as:

(3) $$D_{q}=\frac{\phi_{j}^{T}K_{\sigma }\phi_{j}}{\phi_{j}^{T}K_{0}\phi_{j}}=\frac{\omega_{j}^{2}\phi_{j}^{T}M\phi_{j}}{\phi_{j}^{T}K_{0}\phi_{j}}-1$$

This formulation accounts for the influence of the pre‐stress on the resonator's stiffness and the subsequent effect on the targeted eigenmode.

### Topology Optimization

The method of topology optimization employed here was density‐based,^[^ ^34^ ^]^ which transformed the binary 0‐1 material distribution problem into a continuous optimization problem. The three‐field topology optimization scheme^[^ ^35^ ^]^ defines a design variable ρ_*e*_ ∈ [0, 1] for each element in the FEA mesh, representing the material occupancy of that element. To avoid checkerboard pattern and ensure mesh dependence,^[^ ^20^ ^]^ the design variables were first filtered and then projected to improve discreteness as follows:

(4) $${\tilde{\rho }}_{e}=\frac{\sum_{i\in n_{e}}\left(\rho_{i}v_{i}H_{e,i}\right)}{\sum_{i\in n_{e}}\left(v_{i}H_{e,i}\right)},{\bar{\rho }}_{e}=\frac{\tanh\left(\beta \eta \right)-\tanh\left(\beta ({\tilde{\rho }}_{e}-\eta )\right)}{\tanh\left(\beta \eta \right)-\tanh\left(\beta (1-\eta )\right)}$$

where *n*_*e*_ is the set of neighboring elements whose center‐to‐center distance Δ(*e*, *i*) is smaller than the filter radius $r_{min}=5\mu m$. *H*_*e*, *i*_ is the weight function defined as *H*_*e*, *i*_ = max (0, *r*_*min*_ − Δ(*e*, *i*)), and *v*_*i*_ represents the volume of element *i*. The parameter β controls the sharpness of smoothed Heaviside function and is updated during the optimization process, while η serves as the threshold. ${\bar{\rho }}_{e}$ is the physical density variable used for material distribution. As β increases during the optimization loop, the filtered design variables ${\tilde{\rho }}_{e}>\eta$ are interpreted as Si_3_N_4_ layers with ${\bar{\rho }}_{e}\approx 1$, while ${\tilde{\rho }}_{e}<\eta$ are interpreted as voids with ${\bar{\rho }}_{e}\approx 0$.

The Young's modulus was interpolated using the Rational Approximation of Material Properties (RAMP) method,^[^ ^36^ ^]^ while the material mass density was linearly interpolated:

(5) $$\begin{array}{cc} E_{e} & =\frac{{\bar{\rho }}_{e}}{1+q(1-{\bar{\rho }}_{e})}\left(E_{0}-E_{\min}\right)+E_{\min},q=3\\ \varrho_{e} & =\varrho_{\min}+\left(\varrho_{0}-\varrho_{\min}\right){\bar{\rho }}_{e},{\bar{\rho }}_{e}\in \left[0,1\right] \end{array}$$

where *E*_min _ = 10^−6^ *E*_0_ and ϱ_min _ = 10^−7^ϱ_0_ are used to suppress spurious modes in the eigenfrequency analysis caused by inappropriate stiffness‐to‐mass ratios in low‐density regions. To reduce wrinkling‐like instabilities in low‐density regions, displacement field interpolation was applied as described in Gao et al.^[^ ^21^ ^]^ Instabilities in high‐density regions were controlled by employing mixed formulation elements, where the transnational degrees of freedom (DoFs) were interpolated with quadratic shape functions, while the rotational DoFs were interpolated using linear shape functions. This approach ensured accurate modeling across varying density regions, maintaining stability throughout the optimization process.

To impose length scale constraints on the blueprint design and ensure manufacturability—particularly limited by the resolution of photoresist exposure—a geometry constraint^[^ ^37^ ^]^ was applied. The optimization problem was formulated as follows:

(6) $$\begin{array}{c} \begin{array}{cc} & \max_{\rho_{e}}:D_{q}\left(\phi_{j}\right)\\ & s.t.:K_{0}\left({\bar{\rho }}_{e}\right)U_{0}=F_{0}\left({\bar{\rho }}_{e}\right),\\ & \left(K_{0}+K_{\sigma }\left({\bar{\rho }}_{e},U_{0}\right)-\omega_{j}^{2}M\left({\bar{\rho }}_{e}\right)\right)\phi_{j}=0,\\ & \omega_{j}\geq {\bar{\omega }}_{j},\\ & \omega_{j-1}\leq \left(1-\varepsilon \right)\omega_{j},\omega_{j+1}\geq \left(1+\varepsilon \right)\omega_{j},\\ & \omega_{1}\geq {\bar{\omega }}_{1},\\ & g_{s}\leq \varepsilon ,g_{v}\leq \varepsilon ,\\ & \frac{\sum_{e}\left({\bar{\rho }}_{e}v_{e}\right)}{\sum_{e}v_{e}}\leq \bar{V},\\ & 0\leq \rho_{e}\leq 1 \end{array} \end{array}$$

where ${\bar{\omega }}_{j}$ and ${\bar{\omega }}_{1}$ represent the lower frequency limit for the targeted and fundamental modes, respectively; $\bar{V}$ is the upper limit of volume fraction; *g*_*s*_ and *g*_*v*_ are geometry constraint functions^[^ ^37^ ^]^ for the solid and void phases, respectively; ϵ = 10^−5^ is a small number used to account for numerical errors; and ε = 0.005 is a small positive value that ensures separation between neighboring modes and the targeted mode. This formulation ensured that the design was not only optimized for the desired eigenmode but also remained feasible for fabrication, with appropriate control over dimensions and material distribution.

The geometry constraint was introduced when β is large, and the topology of the design has become relatively well‐defined. However, even with a large β, topology optimization may still result in some elements with intermediate design variables. To address this, a double filter method^[^ ^38^ ^]^ was used prior to applying the geometry constraint. By adjusting the projection threshold defined in Equation (4), intermediate elements can be selectively retained or removed, allowing for the fine‐tuning of design features. The targeted mode was initially selected according to the eigenmode ϕ_*j*_ of initial guess, ensuring that the displacement on the central pad remained out‐of‐plane. As the topology evolved during optimization, ϕ_*j*_ also changed accordingly. To maintain continuity in mode identification, a mode tracing method^[^ ^39^ ^]^ was applied to track the eigenmode profile that most closely matches the one from the previous optimization step. The targeted mode was separated from neighboring modes because, in opto‐mechanical applications, it was preferable for the mode to be distinct; this made it easier to excite and help maintain the desired ϕ_*j*_. The multi‐constraint optimization problem was solved using the method of moving asymptotes (MMA),^[^ ^40^ ^]^ which relied on the gradient information of the objective function and constraints derived using the adjoint method^[^ ^41^ ^]^ and chain rules.^[^ ^42^ ^]^ Due to the FEA discretization, stair‐step features may appear at the boundaries between solid and void regions, potentially leading to local stress concentrations or warping. To mitigate this, the blueprint designs were smoothed by removing convex features and applying a low‐pass filter, following techniques similar to those described in Høj et al.^[^ ^22^ ^]^ This approach ensured smoother final design while preserving the structural integrity and performance of the resonator.

### Fabrication and Characterization

The fabrication process started by depositing a 50nm thick Si_3_N_4_ layer as the resonator material on a 4‐inch silicon wafer, 500μm thick, using low‐pressure chemical vapor deposition (LPCVD). A layer of photoresist was deposited on top of the Si_3_N_4_, followed by selective exposure and development using tetramethylammonium hydroxide (TMAH). The exposed areas were etched away using reactive ion etching (RIE) to define the resonator structure, and the remaining photoresist was removed with oxygen plasma. The wafer was then diced into individual chips, which were mounted onto custom‐designed holders. The resonators were released from the silicon substrate by etching in potassium hydroxide (KOH) at 60°C, followed by cleaning with hydrochloric acid and a solution of Piranha. The samples were dried using ethanol vapor for 20 min to prevent stiction.

For characterization, a 1550 nm laser was directed onto the vibrating resonator, and the resulting phase shift due to the motion was detected with high sensitivity using a phase‐locked homodyne detector. The resonator was driven at its resonance frequency, and once resonance was achieved, the excitation is abruptly switched off. The decay of the resonator's amplitude was recorded, allowing for the evaluation of the the quality factor *Q*.

## Conflict of Interest

The authors declare no conflict of interest.

## Data Availability

The data that support the findings of this study are available from the corresponding author upon reasonable request.
